# Impulse control disorders in Parkinson's: Sleep disorders and nondopaminergic associations

**DOI:** 10.1002/brb3.904

**Published:** 2018-02-10

**Authors:** Samuel Carbunaru, Robert S. Eisinger, Adolfo Ramirez‐Zamora, Dana Bassan, Amin Cervantes‐Arriaga, Mayela Rodriguez‐Violante, Daniel Martinez‐Ramirez

**Affiliations:** ^1^ Department of Neurology Center for Movement Disorders and Neurorestoration University of Florida Gainesville FL USA; ^2^ Instituto Nacional de Neurología y Neurocirugía Universidad Nacional Autónoma de México Mexico City México; ^3^ Tecnologico de Monterrey Escuela de Medicina y Ciencias de la Salud Monterrey Nuevo Leon México

**Keywords:** impulse control disorders, nondopaminergic medications, Parkinson's disease, sleep disorders

## Abstract

**Objectives:**

Impulse control disorders (ICDs) are common among patients with Parkinson's disease (PD). Risk factors identified for developing ICDs include young age, family history, and impulsive personality traits. However, the association of these potentially disabling disorders with nondopaminergic drugs and sleep disorders has been understudied. Our objective was to examine the association between ICDs and nondopaminergic medications and sleep disorders.

**Methods:**

We conducted an observational study of 53 patients with PD from the National Institute of Neurology and Neurosurgery. ICDs were diagnosed using the Questionnaire for Impulsive–Compulsive Disorders in Parkinson's Disease Rating Scale (QUIP‐RS). Patients underwent polysomnography screening to diagnose the presence of sleep disorders. We documented the presence of dopaminergic and nondopaminergic medications, including monoamine oxidase type B inhibitors (MAOBIs), antidepressants, sleep inductors, and antipsychotics.

**Results:**

ICDs were reported in 18.9% of the patients (*n* = 10), and sleep disorders were diagnosed in 81.1% of patients (*n* = 43). 32.1% of the patients were on antidepressants, 17% on MAOBIs, 15.1% on sleep inductors, and 1.9% on antipsychotics. We observed that QUIP‐RS A–D subscore depended on the presence of antidepressants (*p* = .03) and sleep inductors (*p* = .02). Sleep disorders were not associated with the total QUIP‐RS score (*p* = .93) or QUIP‐RS A–D subscore (*p* = .81).

**Conclusion:**

Antidepressants and sleep inductors were significant predictors for individual QUIP‐RS items and subscores. Our results suggest that nondopaminergic drugs commonly used for PD may be associated with impulse control disorders. We did not identify a relationship between ICDs and polysomnography‐confirmed sleep disorders in patients with PD. Larger and longitudinal studies are needed to confirm our results.

## 1 INTRODUCTION

Impulse control disorders (ICDs) are repetitive and reward‐based behaviors commonly observed in patients with Parkinson's disease (PD) (Weintraub et al., 2010). ICDs refer to four major disorders including pathological gambling, hypersexuality, compulsive shopping, and binge eating (Voon & Fox, 2007). Other related impulse control behaviors are classified separately and include punding, hobbyism, hoarding, and dopamine dysregulation syndrome (DDS). Epidemiological studies report an estimated prevalence of 8.1%–35% in patients with PD, and around 14% will report having at least one active ICD (Maloney, Djamshidian, & O'Sullivan, 2017). The underlying etiology for an increased prevalence of ICDs in patients with PD is not fully known. ICDs are commonly attributed to hyperdopaminergic states, resulting primarily from levodopa (LD) and dopamine agonists (DAs) (Ray & Strafella, 2010). These drugs play a large role in reward‐motivated behaviors, and their contribution to the risk of developing ICDs is reported to be at least 2–3.5 times higher with DAs than with levodopa (Weintraub et al., 2010). Other reported risk factors associated with ICDs include young age, smoking, alcohol use, family history of pathological gambling, and having impulsive personality traits (Sharma et al., 2015).

Several studies have explored the possible relationship and underlying mechanisms between impulsivity and sleep disorders in patients with PD (Djamshidian, Poewe, & Hogl, 2015; Scullin et al., 2013). However, these have shown no association between polysomnography (PSG)‐confirmed REM behavioral sleep disorder (RBD) and ICDs (Bayard et al., 2014; Romenets et al., 2012). The role of other PSG‐confirmed sleep disorders in ICDs has not been studied. Furthermore, the role of commonly used nondopaminergic drugs in ICDs has also not been investigated. Identifying additional risk factors associated with ICDs is critical to better understand the pathophysiology of these disorders that may lead to prevention or early management of ICDs. These patients experience significantly lower disability and health‐related quality of life, greater functional impairment, and increased caregiver burden (Maloney et al., 2017). In this study, we aimed to determine the association between ICDs and nondopaminergic drugs and with the presence or absence of sleep disorders.

## 2 METHODS

This is a retrospective study of 53 patients with PD from the National Institute of Neurology and Neurosurgery (NINN)‐Movement Disorders Clinic. Data were collected between June 2009 and May 2013. Patients were diagnosed with PD by a movement disorders‐trained specialist (Hughes, Daniel, Kilford, & Lees, 1992). Patients were included if they performed a complete PSG screening analysis within 1 year of sleep complaints. We documented demographics, disease duration, family history of PD, clinical variables, levodopa equivalent dosage (LED), and dopaminergic medications, including LD, DAs, and monoamine oxidase type B inhibitors (MAOBIs). We calculated LED using the following formula: regular LD dose + LD controlled release dose × 0.75 + LD × 0.33 if entacapone + pramipexole dose × 100 + ropinirole dose × 20 + rotigotine dose × 30 + pergolide dose × 1 + bromocriptone dose × 10 + selegiline dose × 10 + rasagiline dose × 100 + amantadine dose × 1 (Tomlinson et al., 2010). We also documented the presence or absence of nondopaminergic drugs including antidepressants, sleep inductors, and antipsychotics. Clinical variables were measured using the Unified Parkinson Disease Rating Scale (UPDRS) in the “on” medication state and the Hoehn and Yahr (H&Y) scale, both scored by a movement disorders‐trained neurologist. The Questionnaire for Impulse Control Disorders in Parkinson's Disease Rating Scale (QUIP‐RS) was used to screen for ICDs. This scale is validated as screening measure that accurately diagnoses ICDs in PD through a self‐guided questionnaire. The QUIP‐RS consists of an ICD subscore A–D including the four main ICDs (A‐pathological gambling, B‐hypersexuality, C‐compulsive shopping, and D‐binge eating) and a total QUIP‐RS score including A–D subscore plus scores from sections E (hobbyism) and F (punding). The questionnaire also evaluates medication usage; however, it is not included in the total score. Higher scores indicate a higher frequency of behaviors (Weintraub et al., 2009). All subjects completed an overnight PSG exam at the NINN–Sleep Disorders Clinic using Grass Technology Twin Polysomnographer (version 4.5.0.27). Conventional electrodes were placed at specific locations following the international 10–20 system, to ensure standardized reproducibility and to rule out epilepsy. According to the American Academy of Sleep Medicine Manual for the scoring of sleep and associated events, the following variables were recorded: electrography, chin, upper, and lower extremities electromyography, electrooculography, pulse oximetry, and abdominal and chest respiratory effort acquisition. A blinded sleep medicine specialist scored the results in agreement with the International Classification of Sleep Disorders to identify the presence or absence of obstructive sleep apnea (OSA), periodic leg movement disorder (PLMD), and RBD (Duchna, 2006).

### 2.1 Statistical analysis

To investigate the relationship between ICDs, PSG‐confirmed sleep disorders, and nondopaminergic drugs, we developed linear regression models. Dependent variables were either specific QUIP‐RS items, subscores, or QUIP‐RS total score. When appropriate and unless otherwise specified throughout, potential confounding variables were added to all models as independent variables. These included H&Y stage, age, gender, family history, disease duration, and UPDRS Part I, II, III, and IV scores. We used posthoc backward elimination to remove uninformative independent variables. We first analyzed the association between ICDs and scores for OSA, PLMD, and RBD as independent measures. We developed separate models with dependent variables being individual scores from QUIP‐RS as well as section subscores and total scores. To extend these results, the next models we tested included medications as additional independent variables. Importantly, we included both dopaminergic and nondopaminergic drugs as predictor variables to examine the understudied relationship between nondopaminergic drugs and ICDs. Backward elimination was applied to drop uninformative predictors. All analyses were completed using R version 3.2 for Mac.

## 3 RESULTS

Patient demographics and clinical characteristics, ICDs, and sleep variables are shown in Table 1. Thirty‐two (60.4%) patients were female. The mean age was 61.6 years (*SD* = 10.9, range 31–81), and the mean disease duration was 6.6 years (*SD* = 6.8, range 1–43 years). UPDRS “on” medication state scores were as follows: part I of 13.9 (*SD* = 6.0), part II of 14.3 (*SD* = 8.5), part III of 27.7 (*SD* = 14.9), and part IV of 2.4 (*SD* = 3.4). The cohort had a H&Y stage of 2.2 (*SD* = 0.7), with 75.5% classified as stage I–II, 18.9% as stage III, and 5.7% as IV–V. Family history of tremors or PD was reported in 81.1% of the cohort. Nearly, all patients (98.1%) were receiving a dopaminergic drug with 75.5% receiving levodopa, 83.0% receiving DA, and 17% receiving MAOBIs. The LED in our sample was 643.6 mg (*SD* = 432.5, range from 0 to 1927.5 mg). In regard to the presence or absence of nondopaminergic medications, 32.1% were on antidepressants, 15.1% on sleep inductors, and 1.9% on antipsychotics. ICDs were reported in ten patients (18.9%), with five patients (9.4%) having a combination of multiple ICDs. Binge eating was documented in four patients, abnormal sexual behaviors in four patients, pathological shopping in four, and pathological gambling in two patients. Hobbyism–punding was reported in eight patients. PSG results confirmed the diagnosis of sleep disorders in 43 patients (81.1%), where 30 patients (56.6%) were diagnosed with OSA, 26 patients (49.1%) with RBD, and 14 patients (26.4%) with PLMD. Twenty‐two patients (41.5%) were diagnosed as having more than one sleep disorder.

**Table 1:** Demographics and clinical variables of our PD cohort

Variables	Mean
Male, *N* (%)	21 (39.6%)
Age, years (*SD*)	61.6 (10.9)
Disease duration, years (*SD*)	6.6 (6.8)
UPDRS, (*SD*)	
Part I	13.9 (6.0)
Part II	14.3 (8.5)
Part III	27.7 (14.9)
Part IV	2.4 (3.4)
LED mg, (*SD*)	643 (432.5)
Hoehn–Yahr stage, *N* (%)	
Stage 1	6 (11.3%)
Stage 2	34 (64.2%)
Stage 3	10 (18.9%)
Stage 4	3 (5.7%)
Dopaminergic medication, *N* (%)	52 (98.1%)
Dopamine agonist	44 (83.0%)
Levodopa therapy	40 (75.5%)
MAOBIs	9 (17.0%)
Nondopaminergic medication, *N* (%)	
Antidepressants	17 (32.1%)
Antipsychotic	1 (1.9%)
Sleep inductors	8 (15.1%)
ICDs, *N* (%)	10 (18.9%)
Binge eating	4 (7.5%)
Sexual behaviors	4 (7.5%)
Shopping	4 (7.5%)
Gambling	2 (3.8%)
Hobbyism–punding	8 (15.1%)
Combination of ICDs	5 (9.4%)
Sleep, *N* (%)	43 (81.1%)
OSA	30 (56.6%)
RBD	26 (49.1%)
PLMD	14 (26.4%)
Combination of sleep disorders	22 (41.5%)

PD, Parkinson's disease; UPDRS, Unified Parkinson's Disease Rating Scale; LED, levodopa equivalent dosage; MAOBIs, monoamine oxidase type B inhibitors; ICDs, impulse control disorders; OSA, obstructive sleep apnea; RBD, REM behavioral sleep disorder; PLMDs, periodic limb movement disorders.

### 3.1 QUIP‐RS and sleep disorders

Sleep disorders were not statistically associated with total QUIP‐RS score (*p* = .93) or QUIP‐RS A–D subscore (*p* = .81), which only evaluates the four main ICDs (i.e., pathological gambling, hypersexuality, compulsive shopping, and binge eating). After backward elimination, we found that Item 1A of the QUIP‐RS (How much do you think about gambling?) significantly depended on OSA score (β = 0.14, *p* = .02) to result in a model with *R* ^2^ = .11, *p* = .02. Additionally, Item 1F of the QUIP‐RS (How much do you think about punding?) significantly depended on RBD score (β = 0.22, *p* = .03) to result in a model with *R* ^2^ = .09, *p* = .03. All other QUIP‐RS items did not significantly depend on sleep disorder scores. After controlling for possible confounding variables (H&Y stage, age, gender, family history, disease duration, and UPDRS Part I, II, III, and IV scores), OSA score still significantly predicted QUIP‐RS Item 1A (β = 0.14, *p* = .02) without any other significantly contributing variables (*R* ^2^ = .16, *p* = .015). With these additional control variables, RBD score no longer significantly predicted QUIP‐RS Item 1F (β = 0.17, *p* = .07), but for this model (*R* ^2^ = .21, *p* = .02), disease duration became statistically significant (β = 0.04, *p* = .03).

### 3.2 QUIP‐RS and nondopaminergic drugs

**Figure 1:**
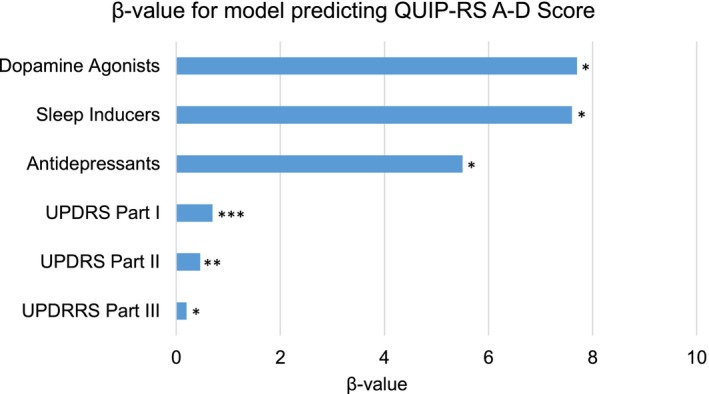
Variables predicting QUIP‐RS A–D Score. Asterisks (*) indicate *p*‐value range of each variable, where *p ≤ .05, **p ≤ .01, and ***p ≤ .005. *Y*‐axis indicates variables used for the model predicting QUIP‐RS A–D score, and *X*‐axis indicates the β‐value obtained from the statistical analysis

We found that QUIP‐RS A–D subscore depended on the presence of DAs (β = 7.7, *p* = .03), antidepressants (β = 5.5, *p* = .03), sleep inductors (β = 7.6, *p* = .02), and UPDRS Part I (β = 0.7, 0.002), Part II (β = ‐0.46, *p* = .01), Part III scores (β = 0.2, *p* = .04). This model as a whole was highly significant (*R* ^2^ = .4, *p* = .009). We also found that total QUIP‐RS score depended only on antidepressant use (β = 7.7, *p* = .04) as well as UPDRS Part I (β = 0.8, *p* = .02) (*R* ^2^ = .16, *p* = .04). Figure 1 shows the significant variables of the model predicting QUIP‐RS A–D subscores.

Individual items from the QUIP‐RS eating section were related to dopaminergic and nondopaminergic drugs. The eating total subscore (Part D) significantly depended (*R* ^2^ = .55, *p* = .002) on the DAs Pramipexole (β = 4.3, *p* = .008) and Rotigotine (β = 3.8, *p* = .04), on MAOBIs (β = 2.6, *p* = .04), and on sleep inductors (β = 3.9, *p* = .002), with significantly contributing control variables being UPDRS Part I (β = 0.27, *p* < .001) and Part III (β = 0.2, *p* = .04). MAOBIs significantly predicted QUIP‐RS Item 3D (Do you have difficulty controlling eating behavior?) (*p* < .001) and eating total subscore (*p* = .04), and sleep inductors significantly predicted QUIP‐RS Items 1D (How much do you think about eating behavior?) (*p* < .001), 2D (Do you have urges or desires for eating behavior?) (*p* = .005), and 3D (Do you have difficulty controlling eating behavior?) (*p* < .001), and total QUP‐RS A–D subscore (*p* = .02), as shown in Table 2.

**Table 2:** Model showing significant associations between nondopaminergic medications and individual QUIP‐RS items

Variables	*p*‐Value
MAOBIs	
QUIP‐RS binge eating subscore	.04
QUIP‐RS 3D score (do you have difficulty controlling eating behavior?)	<.001
Antidepressants	
QUIP‐RS total score	.04
QUIP‐RS A–D score	.03
Sleep Inducers	
QUIP‐RS A–D subscore	.02
QUIP‐RS section D‐binge eating subscore	.002
QUIP‐RS 1D (How much do you think about eating behavior?)	<.001
QUIP‐RS 2D score (Do you have urges or desires for eating behavior?)	.005
QUIP‐RS 3D (Do you have difficulty controlling eating behavior?)	<.001

QUIP‐RS, Questionnaire for Impulse Control Disorders in Parkinson's Disease Rating Scale; MAOBIs, monoamine oxidase type B inhibitors.

Model obtained after controlling for Hoehn & Yahr stage, age, gender, family history, disease duration, and Unified Parkinson Disease Rating Scale.

## 4 DISCUSSION

In the present study, we investigated the association between ICDs with sleep disorders and nondopaminergic medications in a Mexican PD cohort. We observed a significant statistical association between the use of nondopaminergic drugs, specifically antidepressants and sleep inductors, with higher total QUIP‐RS A–D subscores, and of MAOBIs with individual QUIP‐RS eating section subscores. Our results highlight a potential association of nondopaminergic drugs to impulsive behaviors in PD. Consistent data have shown an unquestionable relationship between dopaminergic medications and ICDs (Zurowski & O'Brien, 2015); however, the effect of other commonly used medications in PD has been generally understudied. A few case studies have reported the emergence of ICDs after treatment with MAOBIs. Vitale et al. (2013) reported two cases of patients with PD developing ICDs after being treated with rasagiline. Another case was reported of a patient with PD that developed hypersexuality after being treated de novo with rasagiline (Reyes, Kurako, & Galvez‐Jimenez, 2014). Furthermore, in a multicenter, transversal, retrospective study conducted in Spain, an association between ICDs and concomitant treatment with rasagiline was observed (Garcia‐Ruiz et al., 2014). The role of MAOBIs in ICDs has been linked to an increase in behavioral plasticity and implicated in personality trait such as impulsivity and aggression (Harro & Oreland, 2016). The effects of antidepressants on the emergence of ICDs are less well established. A case of venlafaxine‐induced kleptomania was recently reported (Demartini, D'Agostino, Basi, & Gambini, 2016). In fact, several open‐label studies in non‐PD patients have shown an improvement in impulse control disorders with antidepressants (Black, Shaw, Forbush, & Allen, 2007; Camardese, Picello, & Bria, 2008; Grant & Potenza, 2006; Padala, Madaan, & Sattar, 2007). Although not clearly understood how antidepressants were associated with ICDs in our PD cohort, we hypothesize a similar mechanism than with dopaminergic drugs, which possibly cause a dopaminergic overactivity of the ventral cognitive‐limbic loop of the basal ganglia (Alzahrani & Venneri, 2015). Similarly, no data have been reported in regard to the relationship between sleep inductors and ICDs. Sleep inductors regulate sleep cycles through sedative interactions with GABA receptors located in the cerebral cortex, thalamus, and hypothalamus (Szabadi, 2006). A recent study reported evidence that GABAergic dysregulation is correlated with impulsivity, supporting claims of previous studies that suggest an association between increased impulsivity and GABA modulating drugs, such as benzodiazepines (Hayes et al., 2014; Mick et al., 2017). We also observed a specific association of dopaminergics, antidepressants, and MAOBIs with QUIP‐RS items related with eating behaviors. Other factors not included in the analysis need to be considered before determining this association, such as genetic, environmental, and psychological factors (Saez‐Francas et al., 2016). Furthermore, given that data in this retrospective analysis lack indication of drug use, it is difficult to draw conclusions on the directionality of the association. For that reason, precaution should be taken before making any casual conclusions in the data. There is scarce information on the interactions of nondopaminergic drugs on impulsivity pathways, and the small cohorts of patients that have been previously analyzed limit the applicability of the findings.

Our study did not support a significant association between ICDs and PSG‐confirmed sleep disorder, which contrasts some of the previous studies reporting positive associations between RBD and ICD. We can argue that these studies documented RBD through questionnaires which can provide subjective results (Bellosta Diago, Lopez Del Val, Santos Lasaosa, López Garcia, & Viloria, 2016; Fantini et al., 2015; Kim et al., 2014). In a cross‐sectional study of 944 patients, ICDs were found to be more frequent in PD patients with RBD, especially punding; however, after controlling for age and disease duration, the positive results were no longer significant (Fantini et al., 2015). Likewise, a cross‐sectional study of 216 patients with PD using QUIP‐RS as a measure to diagnose ICDs also found an association between ICDs and RBD (Kim et al., 2014). Similar to our findings, studies in which RBD was confirmed through a PSG analysis have shown no significant associations between studied variables. Two previous studies have reported no association between ICDS and PSG‐confirmed RBD (Bayard et al., 2014; Romenets et al., 2012). Although no association was found between QUIP‐RS scores and sleep disorders, significant associations were observed between QUIP‐RS Item 1A scores (gambling) and the presence or absence of OSA. The QUIP‐1 score relates to the amount of time a patient thinks of the impulsive behaviors, causing them anxiety and guilt. This finding suggests that persistence of thinking about an impulsive behavior more than other activities may hint at the presence of a sleep disorder in patients with PD. The complex nature of sleep limits the ability to accurately measure associations. Although previous studies that used more subjective data on sleep disorders—such as questionnaires—found an association with ICDs, studies that used a PSG to identify the presence of sleep disorders found no association with ICDs (Bayard et al., 2014; Bellosta Diago et al., 2016; Fantini et al., 2015; Kim et al., 2014; Romenets et al., 2012). This discrepancy is probably due to the different screening tools that were utilized by the studies. Using PSGs to detect sleep disorders might provide more accurate data on sleep and enhance the objectivity of the analysis.

Several limitations of the present study should be considered before interpreting results. Our small sample size might not be representative of the PD population. For instance, the age range and disease duration ranges were relatively large. Furthermore, not all sleep disorders and nondopaminergic drugs were studied, nor the clinical indication for taking these drugs or the class of antidepressant, sleep inductors, or antipsychotic used. Future studies with more specific sample characteristics will be necessary to solidify the results presented here. It is also important to mention that the association between nondopaminergic drugs and ICDs could be bidirectional, which cannot be determined by our study that solely focused on drug use irrespective of indications. Our observation might have clinically relevant implications, and judicious use of nondopaminergic medications in ICDs should be considered. It is also important to note that the *R* ^2^ values of the models were generally low, pointing to the multifaceted and complex relationship between ICDs and other factors in patients with PD. Future prospective, controlled studies may overcome some of our limitations. Namely, given the numerous nondopaminergic medications used in the PD population, larger sample sizes are necessary to isolate medications with the highest impact on impulsivity. Additionally, it should be noted that PD patients with ICDs underreport their impulsive natures (Weintraub, 2009). We caution that accurate diagnosis of ICDs is a well‐recognized difficulty that has yet to be easily overcome.

## 5 CONCLUSIONS

Our results suggest that nondopaminergics may be risk factors for developing ICDs in PD. Associations between sleep disorders and ICDs were not observed. Our findings should be further investigated using prospective, longitudinal studies with larger cohorts. Future research is required to reach definitive conclusions on how the pathways between medications and disorders overlap and what effects this may have on impulsivity.

## CONFLICT OF INTERESTS

None declared.
